# Clinical impact of double-faecal immunochemical testing following implementation into standard triage and investigation of primary care referrals in patients with lower gastrointestinal symptoms

**DOI:** 10.1093/bjsopen/zraf098

**Published:** 2025-10-08

**Authors:** Adam D Gerrard, Yasuko Maeda, Colin Noble, Frances Gunn, Lorna Porteous, Rebecca Cheesbrough, Alastair Thomson, Malcolm G Dunlop, Farhat V N Din, Edinburgh Colorectal Group, A Clark, M Collie, D Collins, M Duff, S Goodbrand, J Mander, H Paterson, M Potter, C Reddy, D Speake, F Shaban, G Smith, P Vaughan-Shaw, N Ventham

**Affiliations:** Cancer Research UK Scotland Centre, Institute of Genetics and Cancer, University of Edinburgh, Edinburgh, UK; Department of Colorectal Surgery, Western General Hospital, Edinburgh, UK; School of Medicine, Dentistry and Nursing, University of Glasgow, Glasgow, UK; Department of Surgery, Queen Elizabeth University Hospital, Glasgow, UK; Department of Gastroenterology, Western General Hospital, Edinburgh, UK; Interface Triage Office, Western General Hospital, Edinburgh, UK; Primary Care, NHS Lothian, UK; Primary Care, NHS Lothian, UK; NHS Analytical Services, Western General Hospital, Edinburgh, UK; Cancer Research UK Scotland Centre, Institute of Genetics and Cancer, University of Edinburgh, Edinburgh, UK; Department of Colorectal Surgery, Western General Hospital, Edinburgh, UK; Cancer Research UK Scotland Centre, Institute of Genetics and Cancer, University of Edinburgh, Edinburgh, UK; Department of Colorectal Surgery, Western General Hospital, Edinburgh, UK; Department of Colorectal Surgery, Western General Hospital, Edinburgh, UK

## Abstract

**Background:**

Faecal immunochemical testing has rapidly been established as the first-line triage for patients with symptoms suspicious for colorectal cancer. However, the reported low compliance of test returns issued from primary care is concerning. This article reports the real-world impact of implementation of a double-faecal immunochemical testing pathway for symptomatic referrals into routine clinical practice.

**Methods:**

All eligible referrals between November 2021 and October 2022 were sent two faecal immunochemical tests via the faecal immunochemical testing interface office. Colorectal investigations were instigated if either test result was ≥10 µg haemoglobin per g. Referrals with double-negative results were reviewed by consultants who decided whether symptoms merited further investigation. Cancer registry follow-up data were cross-checked, and a further electronic registry allowed capture of re-referrals.

**Results:**

Some 5425 patients were triaged using double-faecal immunochemical testing, with 5116 (94.3%) completing at least 1 and 4607 (84.9%) both faecal immunochemical tests. The positivity of one test was 20.8%, rising to 27.8% where both tests were completed. The number of referred patients undergoing colorectal investigation fell from 90% before faecal immunochemical testing-directed pathways to 56.6% owing to a reduction in investigating patients with double-negative results. The double-faecal immunochemical testing pathway had a sensitivity of 94.3% for the detection of colorectal cancer, with 37.5% of cancers with a negative first test being detected by the second. Only 3.3% of patients triaged through the double-faecal immunochemical testing pathway were re-referred.

**Conclusion:**

The double-faecal immunochemical testing pathway demonstrated high test return rates, a reduction in unnecessary investigations, and colorectal cancer detection rates similar to preimplementation rates.

## Teaser

A double-faecal immunochemical testing pathway was implemented in secondary care for all patients with symptoms suspicious for colorectal cancer. There were very good return rates of tests, and the workload of colorectal investigations fell as a result of reassurance and watch and wait for double-negative tests. The use of a second test identifies colorectal cancers that would be missed with a single test.

## Introduction

Immunochemical testing for faecal haemoglobin (Hb) has been shown to be superior to traditional clinical high-risk symptoms for risk stratification of patients with symptoms suggestive of colorectal cancer (CRC) and prioritization of lower gastrointestinal (GI) investigation^1,2^. Consequently, faecal immunochemical testing (FIT) has been adopted as the first-line investigation to triage patients’ requirements for further investigation based on the likelihood of underlying pathology.

Guidance from the National Institute for Health and Care Excellence (NICE)^3^, and the Association of Coloproctology of Great Britain and Ireland and the British Society of Gastroenterology^4^, has advised that FIT can be used as a triage tool in primary care, with a positive test (≥ 10 µg faecal Hb per g) triggering referral to secondary care for further investigation. However, meta-analysis^5^ of studies using single FIT (threshold 10 µg Hb per g) reported a pooled sensitivity of 87.2% for CRC diagnosis, highlighting the concerning rate of missed cancers. The authors^6^ previously showed that a double-FIT strategy, with two separate tests a few days apart, is a robust method for improving test sensitivity to 96.6%. Similarly, other studies^7–9^ using repeat FIT have demonstrated both improved diagnostic test prioritization and detection of significant bowel disease, whereas two negative tests are reassuring and avoid invasive investigation.

A high return rate is central to a successful FIT-directed pathway. Compliance issues have been reported from primary care with some test returns as low as 48%^10^. The authors’ previous study^6^ was administered from secondary care, resulting in test return rates of 89.3% for one FIT and 77.1% for both tests. This high return rate, combined with high double-test sensitivity and a collaborative development of the pathway with primary care, resulted in the implementation of the double-FIT pathway as the standard of care for investigating patients with symptoms suspicious for CRC. Here, the first year of the double-FIT pathway being in operation is reviewed, with an evaluation of whether, when used as routine care, the test return rate and diagnostic performance of the pathway perform as predicted from the previous study. The impact of the double-FIT pathway on colonoscopy and computed tomography (CT) colonography workload, and the re-referral rates, was also assessed.

## Methods

As this work formed part of standard routine clinical care, ethical approval was not required.

### Double-FIT pathway

The double-FIT pathway was managed in secondary care by creation of the Interface Triage Office. This office comprised two nursing and two administration staff members, serving approximately 450 referrals per month. Adult patients aged ≥ 18 years, with symptoms suspicious for CRC according to the *Scottish Referral Guidelines for Suspected Cancer*^11^ (repeated rectal bleeding without obvious rectal cause or blood mixed in stool, persistent change in bowel habit, weight loss, and/or abdominal pain with or without unexplained iron deficiency anaemia), referred with urgent or urgent suspected of cancer (USOC) priority from primary care, were managed universally through the pathway. Previous research had shown a substantial prevalence of CRC in the urgent referrals; therefore, it was decided to manage them both in the pathway. The only exceptions were those with a palpable abdominal or rectal mass who were reviewed in the outpatient clinic. Following assessment and triage by office staff of referrals from primary care, the first FIT kit (Minaris Medical, Tokyo, Japan) was sent out on the next working day. Kits included the sampling device along with a pictorial leaflet describing how to perform the test and an explanatory letter for patients on possible outcomes. Contemporaneously with the dispatch of the first FIT kit, an introductory telephone call was made to the patient explaining the test and emphasizing the need for prompt completion and return to their general practice (GP) surgery. Patients were also alerted to the second test that would be sent 4 days later. Tests were sent at an interval of 4 days to ensure that each was performed on a different stool. It is known from previous work^6^ that the level of discordance between repeated tests is not affected by the time between tests, and so 4 days was chosen to ensure that test results could be actioned within referral time limits. These referral time limits also meant that the pathway had to be designed to send two tests to everyone, rather than awaiting the result of the first test. If a test was not received back within 14 days, reminder telephone calls were made to patients to check that their test kit had been received and to encourage participation. Once returned to their local GP surgery, courier collection delivered tests to the UK Accreditation Service-accredited National Health Service (NHS) Tayside Blood Sciences laboratory based in Ninewells Hospital (Dundee) where samples were analysed to ISO15189 standards using an HM-JACKarc analyser (Minaris Medical). Either test with ≥ 10 µg Hb per g was considered positive, and the patient was triaged to investigation. If both test results were < 10 µg Hb per g or no tests had been completed, referrals were passed to the triaging consultant for evaluation of the need for further investigations or correspondence advising safety-netting and a watch-and-wait approach. A Faecal Immunochemical TesTs for Haemoglobin Evaluation Reporting checklist^12^ can be found in *Appendix S1*.

### Study design and participants

The double-FIT pathway was implemented in clinical practice within NHS Lothian in November 2021. All eligible patients referred to the tertiary colorectal service between then and October 2022 were included prospectively for evaluation in the present study. Patient demographics, anaemia (defined by local laboratory reference values of less than 135 g/l in men, below 120 g/l in women), FIT results, and subsequent colorectal investigations were recorded. Registry data from the South East Scotland Cancer Network were interrogated to ensure complete capture of all CRCs. Re-referral data for patients processed via the double-FIT pathway and re-referred to colorectal services were reviewed.

### Statistical analysis

Quantitative values were compared using χ^2^ test, Fisher’s exact test, or Mann–Whitney *U* test. Positive predictive value, negative predictive value, sensitivity, and specificity were calculated with 95% confidence intervals. Analysis was performed using R version 4·0·5 (R Foundation for Statistical Computing, Vienna, Austria) with associated packages and GraphPad Prism (GraphPad Software, San Diego, CA, USA). FIT results were reported based on the first (FIT1) or second (FIT2) test returned. The highest value result a patient returned, including patients to complete only one or both FITs (FITMAX). And the greatest result of patients to complete both FITs according to the protocol (double-FIT strategy).

## Results

### FIT return and positivity rates, and subsequent investigations

**Fig. 1:**
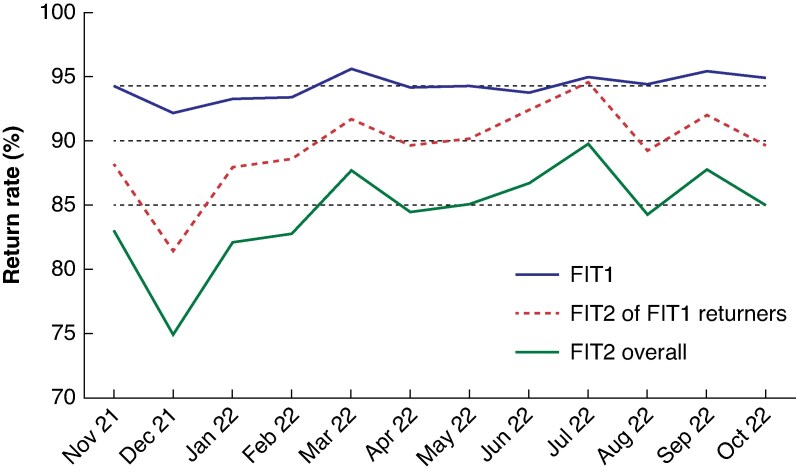
FIT return rates throughout study FIT, faecal immunochemical testing. FIT1, percentage of people sent a test who returned one test; FIT2 of FIT1 returners, percentage of people returning a second test who returned the first test; FIT2 overall, percentage of all people sent a test who returned both tests.

Between November 2021 and October 2022, there were 5425 USOC and urgent referrals of patients with lower GI symptoms who were sent FITs, accounting for 97.2% of all referrals received. The return rate of FIT1 was 94.3% (5116 patients) across the study period; of those who returned FIT1, 90.1% (4607) returned FIT2 (*Fig. 1*). The median time between tests was 5 (interquartile range, i.q.r. 3–7) days. It took patients a median of 3 (1–6) days to complete FIT1 and 5 (1–8) days to complete FIT2. Test positivity at a threshold of 10 µg faecal Hb per g was 20.8% for all patients who returned FIT1 and 27.8% for the double-FIT strategy when two tests were completed, and either result was ≥ 10 µg Hb per g. There was no difference in positivity rates between FIT1 and FIT2 (20.8 *versus* 19.8%; *P* = 0.198). Patient demographics are shown in *Table S1*.

**Fig. 2:**
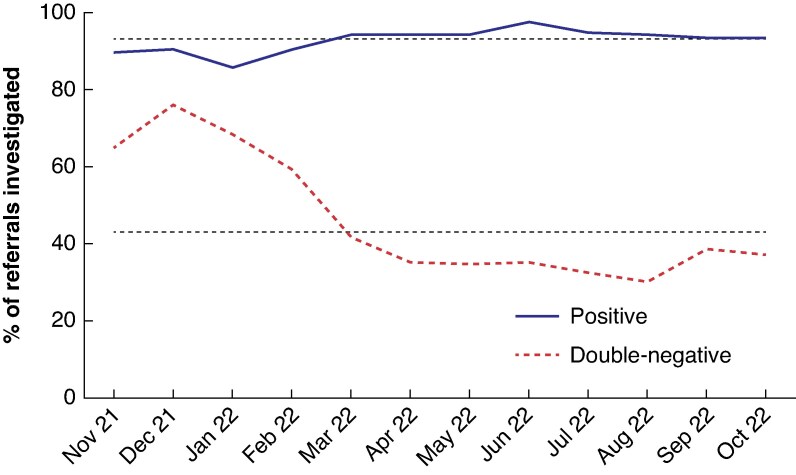
Monthly referrals undergoing colorectal investigation with endoscopy or computed tomography Positive, any faecal immunochemical testing result with ≥ 10 μg haemoglobin (Hb) per g; double-negative, two tests with < 10 µg Hb per g.

Before the double-FIT pathway, 90% of all referrals underwent urgent lower GI investigation with colonoscopy or CT colonography. Over the first year of implementation, the percentage of all referrals investigated fell to an average of 56.6%; this number fell further in the latter months as the pathway became more established and matured in clinical practice. The reduction in the number of people investigated resulted from conscious omission of further diagnostic tests in patients with double-negative FIT, that is those with two test results < 10 µg Hb per g (*Fig. 2*).

### Diagnostic test performance

Over a median follow-up of 16 (i.q.r. 14–20) months (minimum 12 months), 108 CRCs were diagnosed, equating to a 2.0% prevalence in the referred population that was sent FITs. Six of these CRCs were diagnosed in patients who did not return a FIT and, in accordance with the protocol, they were directed to the clinic upon non-return of tests. There was no difference in CRC prevalence between the FIT returners and non-returners (2.0 *versus* 1.9%; *P* = 0.999). Of the 102 with CRC who returned a test, 87 completed both tests. This left 15 patients who completed only 1 test; these 15 patients all had a first test result ≥ 10 µg Hb per g, and so were directed to investigation via the pathway.

The real-world pathway performance in FIT returners based on the intention to double-test, regardless of FIT2 being completed, was assessed. There was a 27.5% positivity rate with 5 CRCs (prevalence 0.1%) where the maximum FIT result was less than 10 µg Hb per g, compared with 97 CRCs (prevalence 6.9%) when the result was ≥ 10 µg Hb per g (*Table S2*). Of those who completed both tests, 712 (15.5%) had discordant FIT results, with 1 test result being ≥ 10 µg Hb per g and the other less, or vice versa. Six patients with CRC (6.9%) had discordant results (*Table 1*). Comparing the first test (FIT1) performance with that of the second test (FIT2) and the combination (FITMAX) for the 4607 patients who completed both tests, in isolation, both single tests, whether FIT1 or FIT2, missed 8 CRCs (*Table 2*). These include five with a double-negative test result, and three each with one negative test and the other positive. This was FIT1-negative, FIT2-positive on three occasions, and the opposite way round for the other three. In the double-test pathway, having either result ≥ 10 µg Hb per g (FITMAX) reduced the percentage of missed CRCs from one test by 37.5% (*Table 1*). The sensitivity for CRC diagnosis at a threshold of 10 µg Hb per g in those completing double-FIT was 94.3 (95% confidence interval 87.1 to 98.1)% at median of 16 months’ follow-up. The effect of different FIT thresholds on positivity and missed CRCs is shown in *Table S3*.

**Table 1:** Double-FIT pathway in 4607 patients completing both tests, FIT results, and CRC diagnosis

First and second FIT result (µg Hb per g)	No. of patients	CRC	CRC prevalence (%)	NNI
< 10, < 10	3327 (72.2%)	5	0.2	665
< 10, ≥ 10	343 (7.4%)	3	0.9	114
≥ 10, < 10	369 (8.0%)	3	0.8	123
≥ 10, ≥ 10	568 (12.3%)	76	13.4	7

Values are *n* (%) unless otherwise stated. FIT, faecal immunochemical test; CRC, colorectal cancer; Hb, haemoglobin; NNI, number needed to investigate.

**Table 2:** Diagnostic performance of the first FIT, second FIT, and double-FIT pathway in 4607 patients completing both tests

	TP	FP	FN	TN	Sensitivity (%)	Specificity (%)	PPV (%)	NPV (%)	Relative reduction in missed CRC (%)
FIT1	79	858	8	3662	90.8 (82.7, 95.9)	81.0 (79.8, 82.2)	8.4 (6.7, 10.4)	99.8 (99.6, 99.9)	–
FIT2	79	832	8	3688	90.8 (82.7, 95.9)	81.6 (80.5, 82.7)	8.7 (7.0, 10.7)	99.8 (99.6, 99.9)	–
FITMAX	82	1198	5	3322	94.3 (87.1, 98.1)	73.5 (72.2, 74.8)	6.4 (5.1, 7.9)	99.8 (99.6, 99.9)	37.5

Values in parentheses are 95% confidence intervals. A positive faecal immunochemical testing (FIT) result is defined as ≥ 10 µg Hb per g. TP, true-positive; FP, false-positive; FN, false-negative; TN, true-negative; PPV, positive predictive value; NPV, negative predictive value; CRC, colorectal cancer. FIT1, first FIT result; FIT2, second FIT result; FITMAX, highest FIT result of two tests.

### FIT-negative CRCs

Five patients with CRC had two FIT results less than 10 µg Hb per g; four of these tumours were American Joint Committee on Cancer (AJCC) stage I cancers, and the fifth was in a patient too frail to undergo resection (*Table S4*). Of the four AJCC stage I cancers, three were in polyps removed at the initial diagnostic colonoscopy and did not require further management, whereas the fourth underwent segmental colectomy. There was a trend towards these double-FIT-negative CRCs being right-sided (*P* = 0.051), but there was no association with anaemia (*P* = 0.646).

### Current CRC detection in comparison with previous years

Given the reduction in the number of symptomatic patients now being investigated because of the double-FIT pathway, assessment was made of how the current number of CRCs diagnosed compared with historical levels to evaluate whether the proportion of expected cancers was being detected from the same population served by NHS Lothian (*Fig. S1*). Accounting for the reduced diagnosis during COVID-19 in 2020 and the subsequent increase in 2021, the volume of CRC diagnoses in 2022 was as anticipated for both overall diagnosis and from primary care referrals.

### Frequency of re-referral from primary care after pathway completion

Of the 5425 patients included in the study, only 177 (3.3%) were re-referred to secondary care colorectal services from primary care. The median time to re-referral was 140 (i.q.r. 60–238) days, with 165 (93.5%) completing one and 150 (84.7%) both FITs at the time of the original referral. Despite a previous decision that re-referred patients would be triaged directly by the consultant of the week, 19 were re-entered into the double-FIT pathway. There was no significant pathology identified in these 19 patients (*Table S5*).

Of the remaining 158 re-referrals not retested, 4 patients ultimately were diagnosed with CRC. The first was to re-request investigations for a patient who was FIT-positive (118 µg Hb per g) but did not attend colonoscopy, whereas a second with positive test results was already awaiting investigation. One man with two undetectable faecal Hb samples was re-referred 129 days later with persistent diarrhoea and was subsequently diagnosed with an AJCC stage I polyp cancer. The final patient had positive test results (400 and 368 µg Hb per g) and owing to frailty underwent CT colonoscopy for loose stools, which did not show any pathology. The patient was re-referred 141 days later with a rectal mass which was confirmed as a cancer. Had the mass been noted in the initial referral, this patient would not have been investigated by the double-FIT pathway but instead directly in the outpatient clinic. None of these cases are considered to be pathway failures, as safety netting and re-referral if persistent symptoms are key properties of the pathway.

### Effect of double-FIT pathway on overall colonoscopy workload

**Fig. 3:**
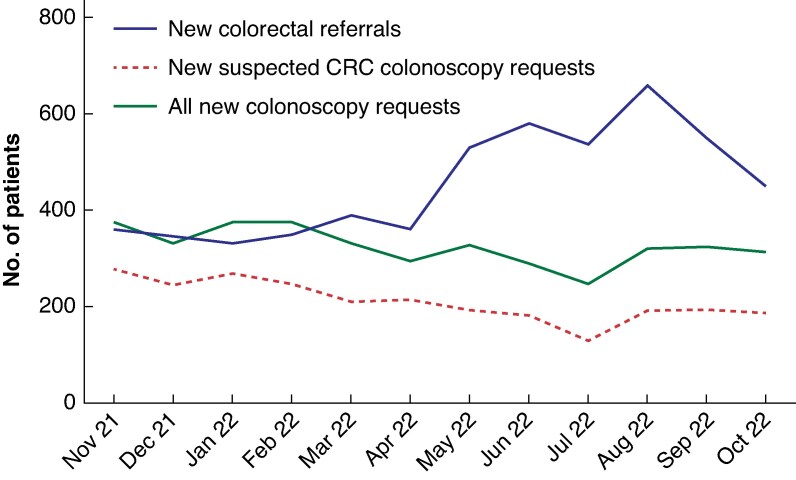
New monthly additions to colonoscopy waiting list CRC, colorectal cancer.

**Fig. 4:**
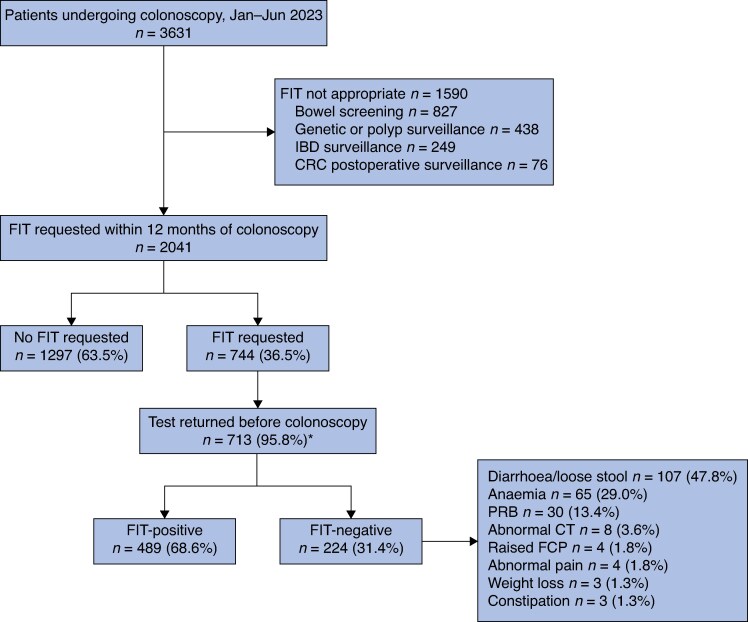
Colonoscopy workload in NHS Lothian between January and June 2023 *Thirty-one patients excluded (744 – 713); patients may have presented with > 1 symptom as reported in the last box on the right. NHS, National Health Service; FIT, faecal immunochemical testing; IBD inflammatory bowel disease; CRC, colorectal cancer; PRB, per rectal bleeding; CT, computed tomography; FCP, faecal calprotectin.

Although symptomatic referrals only form a portion of the overall colonoscopy workload, implementation of this pathway had a notable impact on the total numbers of new patients added to the colonoscopy waiting list. Despite an increase in colorectal referrals over the study period, the number of patients being added to the overall colonoscopy waiting list remained stable (*Fig. 3*). In the first 6 months of 2023, 3631 colonoscopies were performed in NHS Lothian. After removing those undergoing bowel screening, IBD, polyp or genetic surveillance, and patients being followed up after CRC, 744 patients (36.5%) had FIT requested (*Fig. 4*). Of those with a test result, 489 (68.6%) were FIT-positive (≥ 10 µg Hb per g). Of patients in the FIT-negative group who underwent colonoscopy, 29.0% were anaemic.

## Discussion

The performance of the double-FIT pathway in secondary care for patients has been replicated and even surpassed the performance reported in the authors’ previously published work^6^. The triage and investigation of patients with lower GI symptoms suggestive of CRC has fulfilled the expectations, with key performance indicators of return rate and sensitivity being further endorsed by the first year of embedded clinical practice. The pathway has an impressive FIT return rate of 94% for the first test and nearly 85% of all referrals completing both tests. A high return rate is key for FIT-directed pathways to function effectively and safely. Regionally, FIT can only be accessed from secondary care via the purpose-built FIT interface office. The poor return rate of FITs from primary care when accompanying secondary care referrals (48%) has been cited as a contributing factor, cautioning against the use of FIT-directed pathways^10^. By placing FIT in secondary care, the present study has demonstrated the benefit of a dedicated Interface Triage Office. Working closely with the colorectal surgical team, the nurse-led team requests and packages the test kits for postage and contacts the patient to inform them of what to expect and how to complete the tests. Where tests have not been returned within 14 days, the team is contacting patients again to ensure that they have received the test kit and to encourage completion. As this pathway structure has matured, non-return rates in the unit have fallen from 11 to 6%^6^. The CRC prevalence in the non-returners (5.5%) is similar to another reported prevalence of 6.3%^13^. For the pathway to be moved into primary care, a comparable test return rate would need to be achieved. This is unlikely, and so at present centralizing FIT to the interface office in secondary care will continue.

The positivity rate of FIT1 was lower than reported in the initial study of double-FIT (20.8 *versus* 24.8%; *P* < 0.001)^6^. This reduction in the frequency of positive tests returned may reflect changes in the referral behaviours of primary care in the knowledge of initial FIT. Indeed, referral numbers increased by 36% compared with the previous year. The current test positivity is in keeping with the typical 20% reported in other large real-world studies of FIT^1,14,15^.

The double-FIT pathway risk stratifies patients with symptoms that are associated with CRC for further investigation. With a median follow-up of 16 months, of all patients who completed at least one FIT, where the maximum returned test result was < 10 µg Hb per g, the prevalence of CRC was 0.1% compared with 6.9% when the result was ≥ 10 µg Hb per g. Where both test results were ≥ 10 µg Hb per g, the CRC prevalence was 13.4%, emphasizing the need for prioritization of investigation for these patients. The real-world test performance was comparable to that in the authors’ original study^6^. There was diagnostic benefit to the addition of the second test, with three of the eight CRCs with a negative first test being detected by the second. The use of a second FIT has been incorporated into guidelines in both England and Scotland^16,17^, with the reassurance of a low CRC risk in those with double-negative results, allowing risk-managed reduction in investigations^7,18^.

The miss rate of CRC from a single test in this study was 9.2%. As demonstrated in *Table 1*, CRC was missed by a single test, whether that was the first or second test completed. Use of the double-testing strategy mitigated these missed CRCs. The single test missed rate is in keeping with the reported 10.5% in a large FIT study from Nottingham^13^. In that study, the group tried to mitigate this miss rate by combining FIT with blood results and a rectal examination. Here, they reported a 93% sensitivity for CRC detection, although this requires the assumption that all rectal lesions are detected at first examination. The present double-FIT strategy offers comparable sensitivity for a positivity rate of 27.8%, and includes rectal bleeding, a common referral symptom. Enhancing the effectiveness of FIT, reducing missed CRCs while simultaneously reducing unnecessary investigations, is a key future area of research. Personalized risk scores^19^ and the addition of readily available blood tests^20^, along with repeated FIT, may all help achieve more targeted and limited invasive investigations.

The number of referred patients undergoing colorectal investigations has reduced to 57%. This has been driven by a reduction in investigations for patients with two FIT results less than 10 µg Hb per g, which improved as time passed and the pathway matured to now investigate less than 40% of the double-negative referrals. Despite this reduced investigation rate, within the follow-up period, CRC diagnosis has remained as expected, increasing the confidence regarding pathway performance.

Five patients with two FIT results < 10 µg Hb per g were diagnosed with CRC. Investigations were organized for one of these patients owing to persistent symptoms, as part of the safety netting of the pathway; the other four patients had investigations requested based on consultant review of the referral information. The four patients who underwent resection had early polyp cancers that were highly unlikely to be responsible for their symptoms. The fifth patient was anaemic, and, although anaemia was not related to FIT-negative cancers in this study, it has been shown to be associated in other reports^20,21^. Stratified data for patients with anaemia in this study can be found in *Table S6*. A reduction in the threshold of FIT positivity from 10 to 7 µg Hb per g would have seen two of these cancers detected, but would have increased the positivity rate to 31%. Reduction of the definition of FIT positivity to below 10 µg Hb per g has not been considered in the recent NICE guidelines for FIT in CRC care pathways because it would be less cost-effective with reduced reliability as the value approaches the analyser’s limits of quantification^3^.

In the first 6 months of 2023, over one-third of all patients requiring colonoscopy for symptomatic assessment had completed FIT. This mainly represents patients from the CRC referral pathway, and a substantial number of these were FIT-negative (31.4%). Most of these patients were either anaemic or had diarrhoea. Although patients with persistent symptoms may require investigations regardless of the FIT result, additional improvements could be made to reduce unnecessary endoscopy demand further. These include expanding double-FIT for all lower GI symptomatic referrals regardless of referral priority or specialty route, and further scrutiny of whether FIT-negative patients need colonoscopy. The latter will likely reduce as confidence in FIT as a risk stratification tool for significant bowel pathology grows.

A major strength of this study is that it describes the real-world implementation of a new clinical pathway, demonstrating the high return rates that can be achieved in secondary care and the expected performance of double-FIT as both a triage and diagnostic tool. The main weakness is that because not all patients with double-negative results undergo colorectal investigations, the true false-negative rate cannot be known until at least 24 months. The median follow-up so far is 16 months, and the process of reassessment for CRC presentation is ongoing. The positioning of FIT in secondary care in this study may mean it is not generalizable to FIT used in primary care; however, the authors have highlighted some advantages of positioning FIT in secondary care services. Successful implementation requires investment in Interface Triage Office staff, but this is considerably offset by the savings made from avoiding unnecessary investigations.

An effective FIT-directed pathway requires a high rate of compliance with test completion and confidence in the test performance. Double-FIT in secondary care has delivered this over the first year of clinical practice, diagnosing an expected number of CRCs while reducing the demand on investigation services.

## Collaborators

A. Clark, M. Collie, D. Collins, M. Duff, S. Goodbrand, J. Mander, H. Paterson, M. Potter, C. Reddy, D. Speake, F. Shaban, G. Smith, P. Vaughan-Shaw, N. Ventham (Western General Hospital, Edinburgh, UK).

## Supplementary Material

zraf098_Supplementary_Data

## Data Availability

Summary-level data are available on request.

## Supplementary material

*Supplementary material is* available at *BJS Open*  online.
