# Supplementary material for: Clinical impact of double-faecal immunochemical testing following implementation into standard triage and investigation of primary care referrals in patients with lower gastrointestinal symptoms
Source: BJS Open. 2025 Oct 8;9(5):zraf098. doi: 10.1093/bjsopen/zraf098 (PMC12507089; doi:10.1093/bjsopen/zraf098)
Supplement: zraf098_Supplementary_Data [file zraf098_supplementary_data.docx]

**Clinical Impact of Double Faecal Immunochemical Testing Following Implementation into Standard Triage and Investigation of Primary Care Referrals with lower GI symptoms**

Authors

Adam D Gerrard^1,2^, Yasuko Maeda^3,4^, Colin Noble^5^, Frances Gunn^6^, Lorna Porteous^7^, Rebecca Cheeseborough^8^, Alastair Thomson^9^, Malcolm G Dunlop^1,2,10^, Edinburgh Colorectal Group^2^, Farhat VN Din^1,2^

^1^ *Cancer Research UK Scotland Centre, Institute of Genetics and Cancer, University of Edinburgh, Edinburgh, UK*

^2^ *Department of Colorectal Surgery, Western General Hospital, Edinburgh, Scotland*

^3.^ *School of Medicine, Dentistry and Nursing University of Glasgow*

^4.^ *Department of Surgery, Queen Elizabeth University Hospital, Glasgow, Scotland*

^5.^ *Department of Gastroenterology, Western General Hospital, Edinburgh, Scotland*

^6.^ *Interface Triage Office, Western General Hospital, Edinburgh, Scotland*

^7.^ *Lead GP for cancer and palliative care, NHS Lothian, Scotland*

^8.^ *Lead GP for Referrals Advisor, NHS Lothian, Scotland*

^9.^ *Analytical Services, Western General Hospital, Edinburgh, Scotland*

^10.^ *UK Colon Cancer Genetics Group, Medical Research Council Human Genetics Unit, Medical Research Council Institute of Genetics & Cancer, Western General Hospital, The University of Edinburgh, Edinburgh, UK*

**Corresponding author.** Name and address **ORCID ID**; **Twitter**

Farhat Din Bowel Cancer UK-Royal College of Surgeons of Edinburgh Chair of Colorectal Cancer Research

Address: Cancer Research UK Scotland Centre, Institute of Genetics and Cancer, University of Edinburgh, Crewe Road, Edinburgh, UK, EH4 2XU

Email: [Farhat.Din@ed.ac.uk](mailto:Farhat.Din@ed.ac.uk)

ORCID: 0000-0001-5466-8282

**Supplementary Materials - Index**

| **Supplementary Appendixes** |  |
| --- | --- |
| FITTER Checklist | *page 3* |
| **Supplementary Figures and Tables** |  |
| Figure 1: Cases of CRC overall and from primary care referral by year | *page 4* |
|  |  |
| Table 1: Demographics of patients sent FIT tests  . | *page 5* |
| Table 2: Diagnostic performance of the double-FIT pathway in patients who returned at least one FIT | *page 6* |
| Table 3: Effect of different FIT thresholds on positivity and missed CRC in patients who returned two FIT (n=4607, total number of CRC=87) | *page 7* |
| Table 4: Double-FIT negative CRCs | *page 8* |
| Table 5: Re-referrals entered into the double-FIT pathway | *page 9* |
| Table 6: Stratified data for patients with anaemia | *page 10* |

**Supplementary Appendixes**

**Appendix 1: FITTER Checklist**

*Specimen collection and handling*

Between November 2021 and October 2022 all patients referred to secondary care (5,425) were sent two collection devices, median 5 days apart (Minaris Medical Co. Ltd). The device collects 2mg of faeces with a probe attached to the cap into 2.0ml of buffer. Participants pass the probe into the stool, wrote the date of collection, and returned the collection kits to their local primary care centre who transported the kits to the regional laboratory (Dundee, Scotland) where the UKAS accredited NHS Tayside Blood Sciences laboratory is based in Ninewells Hospital. In a timely fashion samples were analysed to ISO15189 standards. In total, 5116 patients completed at least one FIT.

*Analysis*

Specimens were analysed using the HM-JACKarc analyser. Samples were stored at 4°C until the time of analysis and allowed to warm to room temperature. The analytical working range in 7-400µg Hb/g.

*Quality management*

All analysis is carried out at the regional laboratory. There are daily internal quality control measures with repeated sampling and between batch impression aiming for ±2SD. External quality assessment is performed three times per month with sample material prepared by EQU laboratories (Birmingham, England).

*Data Handling*

The f-Hb concentrations are recorded electronically and linked back to the patient record to be available to the clinical team.

**Supplementary Figures and Tables**

**Figure 1: Cases of CRC overall and from primary care referral by year**

**Table 1: Demographics of patients sent FIT tests**

|  | Patients Sent FIT  (n=5425) |
| --- | --- |
| Age (IQR) | 63 (54-74) |
| Sex, Female *(%)* | 3161 *(58.3)* |
| Median SIMD (IQR) | 4 (2-5) |
| Anaemia *(%)* | 933 *(17.2)* |
|  |  |
| FIT1 Returned *(%)* | 5116 *(94.3)* |
| FIT1 Positive *(%)* | 1066 *(20.8)* |
| FIT2 Returned *(%)* | 4607 *(84.*9) |
| FIT2 Positive *(%)* | 911 *(19.8)* |
| **Double-FIT Strategy Positive *(%)*** | **1280 *(27.8)*** |
| **FITMAX Positive *(%)*** | **1409 *(*27.5)** |
|  |  |
| Time Between Tests (Days, IQR) | 5 (3-7) |

SIMD; Scottish Index of Multiple Deprivation, Double-FIT Strategy Positive; Number of patients to complete two FITs with at least one test ≥10µg Hb/g, FITMAX Positive; Number of people to return only one or two tests and have at least one test ≥10µg Hb/g.

**Table 2: Diagnostic performance of the double-FIT pathway in patients who returned at least one FIT**

| **Maximum FIT (µg Hb/g)** | **Number of Patients** | **CRC** | **CRC Prevalence** | **NNI** |
| --- | --- | --- | --- | --- |
| <10 | 3707 (72.5%) | 5 | 0.1% | 741 |
| ≧10 | 1409 (27.5%) | 97 | 6.9% | 15 |

CRC; Colorectal cancer, NNI; Number needed to investigate.

**Table 3: Effect of different FIT thresholds on positivity and missed CRC in patients who returned two FIT (n=4607, total number of CRC=87)**

| FIT threshold (µg Hb/g) | Positivity with single FIT | Positivity with double FIT | CRC missed single FIT | CRC missed with double FIT | Percentage reduction in missed CRC by second test |
| --- | --- | --- | --- | --- | --- |
| 10 | 20.3 % (937) | 27.8% (1280) | 9.2% (8) | 5.7% (5) | 37.5% |
| 20 | 15.8% (727) | 21.9% (1010) | 13.8% (12) | 9.2% (8) | 33.3% |
| 40 | 11.7% (538) | 16.4% (755) | 19.5% (17) | 11.5% (10) | 41.2% |
| 80 | 8.7% (400) | 12.2% (562) | 25.3% (22) | 17.2% (15) | 31.8% |
| 100 | 8.1% (372) | 11.1% (513) | 27.6% (24) | 20.7% (18) | 25.0% |
| 150 | 6.9% (318) | 9.7% (448) | 36.8% (32) | 24.1% (21) | 34.4% |

**Table 4: Double-FIT negative CRCs**

| **Age** | **Sex** | **FITs**  **(µg Hb/g)** | **Anaemia** | **CRC Location** | **AJCC Stage** | **Treatment** |
| --- | --- | --- | --- | --- | --- | --- |
| 58 | Male | 0,7 | No | Sigmoid colon | 1 | Polyp cancer,  endoscopic resection |
| 73 | Female | 0,0 | No | Hepatic flexure | 1 | Polyp cancer,  endoscopic resection |
| 73 | Male | 0,0 | No | Hepatic flexure | 1 | Polyp cancer,  endoscopic resection |
| 75 | Female | 0,0 | No | Transverse colon | 1 | Polyp cancer,  Extended right hemicolectomy |
| 82 | Female | 9,0 | Yes | Ascending colon | - | Palliation - not fit for surgery |

CRC; Colorectal cancer, AJCC; American joint committee on cancer

**Table 5: Re-referrals entered into the double-FIT pathway**

NR; Not Returned, NAD; No Abnormality Detected, DNA; Did Not Attend, OPD; Outpatient Department Appointment

**Table 6: Stratified data for patients with anaemia**

A. Patients with anaemia who have completed two FITs

|  | Number of patients | CRC |
| --- | --- | --- |
|  | 864 | 41 *(4.7%)* |
| First FIT (µg Hb/g) |  |  |
| <10 | 575 *(66.6%)* | 2 *(0.3%)* |
| ≧10 | 289 *(33.4%)* | 39 *(13.5%)* |
| Two FITs (µg Hb/g) |  |  |
| Both FIT <10 | 499 *(57.8)* | 1 *(0.2%)* |
| Any FIT ≧10 | 365 *(42.2)* | 40 *(11.0%)* |

B. Comparison of CRC diagnosed in anaemic vs non-anaemic patients

|  | Anaemia | No Anaemia |
| --- | --- | --- |
| Patients completed two FITs | 864 | 3954 |
| *CRC (Prevalence)* | 41 *(4.7%)* | 59 *(1.5%)* |
| CRC with both FIT <10µg Hb/g *(Prevalence)* | 1 *(0.1%)* | 4 *(0.1%)* |
| Location within colon - Right side: Left side | 1:0 | 3:1 |

Anaemia defined by local laboratory reference values of less than 135g/l in men, below 120g/l in women)
